# Two Dentists Who Do “Not Get On”

**Published:** 1887-12

**Authors:** W. Geo. Beers

**Affiliations:** Montreal


### TWO DENTISTS WHO DO NOT "GET ON."

BY W. GEO. BEERS, L. D. S., MONTREAL.

A good many honest dentists do not get on in the world if they have any rivalry. They wonder at it too, especially when they see the prosperity of men who have nothing but their cheek and assurance to back them. They are temperate; they do not even use tobacco. They go to church, sing in the choir, take up the collection—all, perhaps, from the purest motives. They get notoriety, but not patients. They are not hypocrites; they do not make long prayers and pious pretensions, but they use and enjoy all the collateral advantages of religious society useful to business, by having sittings in several churches of different denominations, accommodating themselves to each one with remarkable facility. I would rather believe these men sincere than hypocritical. It suits the temperament of some to go in heartily for every stir, and to mix in every popular movement. They would do it if they were not dentists, and cannot see why their profession should prevent them.

In spite of the temptations to worldliness, we have many real saints in dentistry, men who instinctively walk in the paths of righteousness, and who have none of the difficulty the rest of us encounter in battling every day and hour with the world, the flesh, and the devil. But, as in every other profession, we have men who take ten times more trouble to get friends and patients than to get knowledge. They live in a fever of unrest, unless every day brings its new acquaintance; but they are content with the experience gained in the past, with the occasional scraps picked up by the way. I am impelled to these sentiments by a conversation I once had with just such a friend as I have described. Like the rest of us, he has his failings, but unlike those of some of us, they lean strongly to virtue's side. Yet he did not succeed in his profession, while he saw ignorant arrogance and assumption forging ahead of him, and younger men recognized at home and abroad as peers of the worthiest.

"Shall I become a quack?" he said to me one day. "If I were to lie, and deceive, and brag, my rooms would be filled by credulous yet confiding patients. Any man of assumption, no matter how ignorant, can make money that way in dentistry, though in the long run he is found out."

"You are on the wrong tack," I replied. "As a crowd is not company, neither is a large practice the true index of success. You could not play the quack or fraud if you tried. But just look at your office. You have not a modern instrument in it; you actually stick to the old key of Garrangeot; you extract all abscessed teeth; you devitalize all exposed pulps; you never extirpate the dead pulp; your only articles of the materia medica are arsenic, creosote and gum sandarac; you have neither a dental engine nor any of the improvements of operative dentistry since the days of Chapin A. Harris; you do not use the rubber dam; you know nothing of the revolution in dental pathology. In fact, you are a professional Rip Van Winkle, and you waken to wonder why the boys, and even the quacks, are going ahead of you. Public opinion will not stand still because you do. It will revere your integrity, but it will not patronize your stagnation. You must begin at the foot again and make up lee-way. You must read our journals, our text-books, and sit, Samuel-like, at the feet of any honest and able brother who will bother himself for your sake. Many a time they have bothered themselves for me and my ignorance. To me the greatest heroes are those of my own profession, who have made conquests in art and science which they willingly share with their brethren."

I converted that old fossil. He goes about now with a heart hungry for information, as if he were born again, and was in his teens in dentistry. People have observed the change. He is healthier as well as happier, and has come to the conclusion that a man's good conduct and unimpeachable honor are no excuse for his professional stagnation.

Just over the way from him another *confrère* never "gets on," simply because he is a filthy hog. If I were a patient I would prefer to trust my mouth to the hands of an ignoramus rather than those of a dentist who is dirty. He is unclean in his person. You can see the high-water mark around his neck, the "lick-and-a-promise" around his ears. His nails have not been out of mourning for the dirt of his fingers since he was weaned. His moustache and whiskers are tell-tales of what he had for breakfast. His hair is full of dandruff, and seldom brushed. To the custom of wearing clothes, his patients are indebted for utter ignorance as to the condition of his skin. From the dirty shirt collar and front to his boots covered with mud, his clothes are bespattered with tobacco juice and plaster of Paris. His towels and napkins are shabby and soiled, and the head-rest of his chair is a musty mystery. His instruments lie uncovered and uncleaned, rusty from dust and the debris of dead dentine. You will be sure to see bloody forceps with the teeth they extracted still in their grip, as if he imagined it was an æsthetic exhibition. His spittoon is dirtier than any *shebeen* sink; old cigar stumps lie upon his operating stand; loafers, perhaps, hang about his rooms, spitting on the stove, with their heels up, to add the perfume of old leather to the incense about them. Dirty, tattered and vulgar illustrated papers, low, sensational literature, cheap and nasty books, coarse pictures mark the personal taste and character of the man. He is intensely one of those unpurified human hogs whom no cannibal would touch with a forty foot spoon, not even if he were boiled to the bones in pepper and brimstone sauce. His mind is just as impure as his body. He is low and slangy in conversation with ladies, vulgar and blasphemous with men. When skunks need human dentists, he may find occupation; but one sight and smell of him is enough.

This picture is not exaggerated. Happily, it is rare. But there are points in it that will prick the conscience of men who do get on, yet whose patients wish them all sorts of prosperity *with a little more taste*. John Wesley struck a key-note of success in dentistry when, in his sermon "On Dress," he emphasized the fact that "Cleanliness is, indeed, next to godliness."

